# Effects of Soybean Agglutinin on Intestinal Barrier Permeability and Tight Junction Protein Expression in Weaned Piglets

**DOI:** 10.3390/ijms12128502

**Published:** 2011-11-29

**Authors:** Yuan Zhao, Guixin Qin, Zewei Sun, Dongsheng Che, Nan Bao, Xiaodong Zhang

**Affiliations:** 1 Key Laboratory of Animal Production, Product Quality and Security, Ministry of Education, Jilin Agricultural University, Changchun 130118, China; E-Mails: zhaoyuan4CL52@126.com (Y.Z.); chedongsheng@163.com (D.C.); baonan203@163.com (N.B.); 2 College of Animal Science and Technology, Jilin Agricultural University, Changchun 130118, China; E-Mail: sunzewei@jlau.edu.cn; 3 Laboratory of Infectious Diseases, College of Animal Science and Veterinary Medicine, Jilin University, Changchun 130012, China; E-Mail: zhang_xd@jlu.edu.cn

**Keywords:** soybean agglutinin, tight junction, intestinal permeability, ZO-1, occludin, piglets

## Abstract

This study was developed to provide further information on the intestinal barrier permeability and the tight junction protein expression in weaned piglets fed with different levels of soybean agglutinin (SBA). Twenty-five weaned crossbred barrows (Duroc × Landrace × Yorkshire) were selected and randomly allotted to five groups, each group with five replicates. The piglets in the control group were not fed with leguminous products. 0.05, 0.1, 0.15 and 0.2% SBA was added to the control diet to form four experimental diets, respectively. After the experimental period of 7 days (for each group), all the piglets were anesthetized with excess procaine and slaughtered. The d-lactic acid in plasma and the Ileal mucosa diamine oxidase (DAO) was analyzed to observe the change in the intestinal permeability. The tight junction proteins occludin and ZO-1 in the jejunum tissue distribution and relative expression were detected by immunohistochemistry and Western Blot. The results illustrated that a high dose of SBA (0.1–0.2%) could increase the intestinal permeability and reduce piglet intestinal epithelial tight junction protein occludin or ZO-1 expression, while low dose of SBA (0.05% of total diet) had no significant affects. The contents of DAO, d-lactic acid, occludin or ZO-1, had a linear relationship with the SBA levels (0–0.2%) in diets. The high dose SBA (0.1–0.2%) could increase the intestinal permeability and reduce piglet intestinal epithelial tight junction protein occludin or ZO-1 expression, while low dose of SBA (0.05% of total diet) had no affects.

## 1. Introduction

Lectins, a kind of glycoprotein compound, can bind to receptors of intestinal epithelial cells [1]. Further effects of lectins include changing the gut immune function, reducing production of endocrine cells and gut hormones, disordering the bacterial balance in the gut lumen, and damaging mucosal cells [2]. Lectins are widely distributed in grain legumes such as faba beans, peas, soybeans and lupins [3–6].

Soybeans are widely utilized in the food and feed industries due to their high nutritional value [7], though they are also limited owing to the occurrence of the anti-nutritional factors (ANFs). Soybean agglutinin (SBA), being one of the major ANFs, is resistant to digestive enzymes in the gastrointestinal tract, which has a unique property of binding to carbohydrate-containing molecules. SBA binds to the intestinal epithelium, which leads to the disruption of the brush border [8] and extends negative effects on the growth of spleen and kidneys [9]. SBA, a glycoprotein, is composed of a tetramer with 30 kDa subunits. Each subunit has a carbohydrate-binding site, with a high affinity for *N*-acetyl-d-galactosamine (GalNAc) [10].

The tight junction located in the apical side of epithelia, a major mode of cell-cell adhesion, plays an important role in maintaining cell polarity and regulating permeability, as a barrier preventing bacteria, endotoxin and toxicity macromolecule crossing an epithelial sheet between adjacent cells. The tight junction is mainly composed of transmembrane proteins (occludin, claudin, and junctional adhesion molecules) and peripheral membrane proteins (zonula occludens [ZO]-1, ZO-2, ZO-3, MUPP-1) [11], and its quantity and distribution decide the efficacy of cell-cell adhesion. Weaning increases the intestinal barrier permeability, and decreases the tight junction protein expression contents of occludin and ZO-1 for piglets [12,13]. Weaned piglets frequently suffer a serious threat to their health from ANFs, including SBA. However, the effects of SBA on intestinal barrier permeability and function are not yet fully elucidated to our knowledge.

This study was developed to provide further information on the intestinal barrier permeability and the tight junction protein expression in weaned piglets fed with different levels of SBA, which are essential criteria to explore the mechanism of SBA-injured intestinal barrier function.

## 2. Results and Discussion

### 2.1. Purification of Glycinin and β-Conglycinin

Purified SBA samples containing more than 95% SBA were kindly provided by Dongsheng Che of Jilin Agricultural University.

### 2.2. Experimental Design

Before weaning, six litters of piglets as optional piglets were fed with dry whole milk at the age of 7-days in order to complement the nutrition and ensure the piglets eat solid feed. Moreover, the diets devoid of soybean protein were used before experiments to avoid the experimental effects from oral tolerance of soybean. All of the piglets were weaned on the 28th day after birth.

Twenty-five weaned crossbred barrows (Duroc × Landrace × Yorkshire) with an average initial body weight of 7.07 ± 0.65 kg were selected in this experiment. After 3 days of adaptation, the piglets were randomly allotted to five groups, each group with five replicates. The piglets in the control group were fed without ingredients originating from leguminous products. 0.05, 0.1, 0.15 and 0.2% SBA was added to the control diet in order to form four experimental diets, respectively. The experimental period was 7 days. The composition and nutrient content of the control diet are shown in Table 1. The diet was formulated to meet NRC (1998) requirements. After the experimental period, all the piglets were anesthetized with excess procaine and slaughtered before the morning meal.

### 2.3. Determination of d-Lactic Acid in Plasma

Blood samples were collected in tubes containing EDTA and mixed immediately to avoid coagulation. Plasma were obtained after centrifugation at 3,000 × g for 15 min at 4 °C and then stored at −80 °C until analysis. The levels of d-lactic acid in plasma were determined by Porcine d-lactic acid ELISA kit (E05227, R&D Systems, Minneapolis, MN, USA).

### 2.4. Determination of Diamine Oxidase (DAO) in Ileal Mucosa

Mucosa were scraped from ileal tissue and then frozen in liquid nitrogen. Frozen mucosa prepared in PBS (0.1 M, pH 7.2) were homogenized and centrifuged at 10,000 × g for 30 min at 4 °C. The supernatants were collected to analyze the diamine oxidase (DAO) according to Li *et al.* (1996) [14].

### 2.5. Immunohistochemistry

Mid-jejunum fragments obtained from control and SBA-treated piglets were rinsed in PBS, fixed in 4% formalin, embedded in paraffin, cut into 5-μm-thick sections and deparaffinized. To detect the tissue distribution of tight junction proteins, a labeled Streptavidin-Peroxidase complex methods (Ultrasensitive Kit, KIT9706, Maixin, Fuzhou, China) was used to bind occludin (bs-1495R, Bios, Beijing, China) or ZO-1(bs-1329R, Bios, Beijing, China). Color was developed in a 0.05% diaminobenzidine (DAB) substrate solution. The sections were then counterstained with hematoxylin, dehydrated, cleared, and permanently mounted. Intestinal sections from control and SBA-treated piglets were stained in parallel.

### 2.6. Immunoblotting

Mid-jejunum tissue specimens obtained from control and SBA-treated piglets were frozen in liquid nitrogen. Frozen tissue samples were washed with PBS (0.01 M). The samples were then lysed on ice in a Potter tissue grinder with lysis buffer (20 nM Tris-HCl pH 8.0, 5 mM EDTA, 1% Triton × 100) supplemented with protease inhibitor cocktail (Cat. 539134, Merck, Darmstadt, Germany). Solution was homogenized, through a 26G needle and sonicated for 3 times 20 s. Homogenate was centrifuged at 10,000 × g for 30 min at 4 °C. Protein quantification of supernatant was determined using the bicinchoninic acid (BCA) protein assay by the microplate procedure. 40 μg of total proteins from tissues was separated by SDS-PAGE and transferred onto PVDF membranes. The membranes were blocked with 3% BSA in PBS-Tween and incubated with rabbit anti-occludin (bs-1495R, Bios, Beijing, China) or rabbit anti-ZO-1(bs-1329R, Bios, Beijing, China) and rabbit anti-β-actin (bs-0061R, Bios, Beijing, China) antibodies diluted 1:1,000, 1:1,000 and 1:2,000 in PBS-Tween-SBA respectively. After washing, they were incubated with 1:10,000 horseradish peroxidase-conjugated anti-rabbilt IgG. After thorough washing, the Superstar Enhanced chemiluminescent kit (AR1111, Boster, Wuhan, China) was applied for antibody detection with X-ray film. Membrane actified solution (AR0153–50, Boster, Wuhan, China) was used to clear the existing antibodies and chemiluminescence in order to rebind another interest protein in the same membrane.

Signal intensities were estimated using Image-Pro Plus 5.0 software. Western blot band density was compared to β-actin in each lane as a loading control.

### 2.7. Statistical Analysis

All data were analyzed using the general linear model procedure of Statistical Package for Social Sciences version 11.5 (SPSS Inc., Chicago, IL, USA). The results were expressed as mean values ± Standard Deviation (SD). Duncan’s multiple range tests was employed to test the differences among the means. Linear regression with calculation of the correlation factor (r) was used for jointly distributed variables. Statements of statistical significance were based upon P ≤ 0.05.

## 3. Results

### 3.1. d-Lactic Acid in Plasma and DAO in Ileal Mucosa

**Figure 1:**
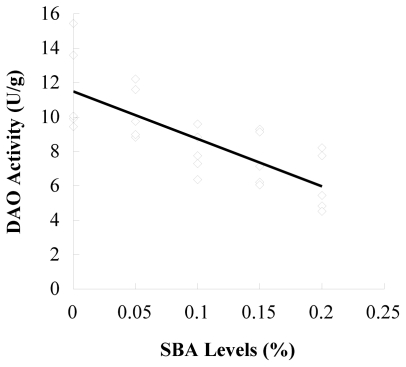
Linear correlations between DAO activity and SBA levels. n = 5; y = −27.57x + 11.49; R^2^ = 0.577; *P* < 0.001.

**Figure 2:**
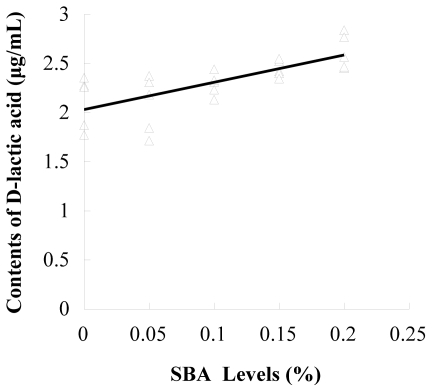
Linear correlations between contents of d-lactic acid and SBA levels. n = 5; y = 2.77x + 2.03; R^2^ = 0.517; *P* < 0.001.

The results of d-lactic acid in plasma and DAO in ileal mucosa determination are shown in Table 2. Ileal mucosa DAO in piglets fed 0.1, 0.15 and 0.2% SBA decreased compared to the control and 0.05 SBA group (*P* = 0.001). The plasma d-lactic acid enhanced with the increase of SBA levels (*P* = 0.002). The contents of DAO (*P* < 0.001, R^2^ = 0.577) or d-lactic acid (*P* < 0.001, R^2^ = 0.517) had linear relationship with the SBA levels (0–0.2%) in diets (Figures 1 and 2).

### 3.2. Occludin or ZO-1 Expression in Mid-Jejunum Tissue

**Figure 3:**
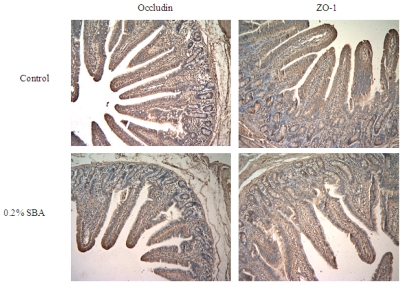
Location of tight junction protein ZO-1 and Occludin in jejunum of weaned piglets. Immunohistochemistry staining (magnification 100×) represents control group and 0.2% SBA group.

**Figure 4:**
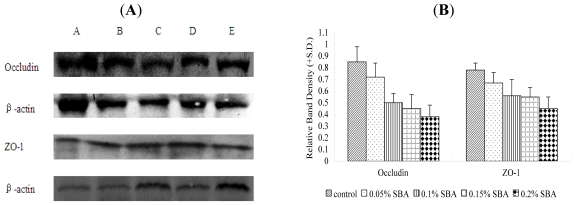
Expression of tight junction protein Occludin and ZO-1 in jejunum of weaned piglets. The tight junction protein Occludin and ZO-1 from jejumun was analyzed by Western-Blot (**A**), and statistically calculated (**B**). The β-actin is housekeeping protein. The data represents different groups.

**Figure 5:**
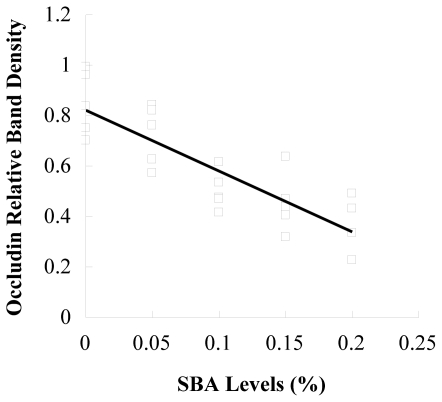
Linear correlations between occludin relative band density and SBA levels. n = 5; y = −2.42x + 0.82; R^2^ = 0.715; *P* < 0.001.

**Figure 6:**
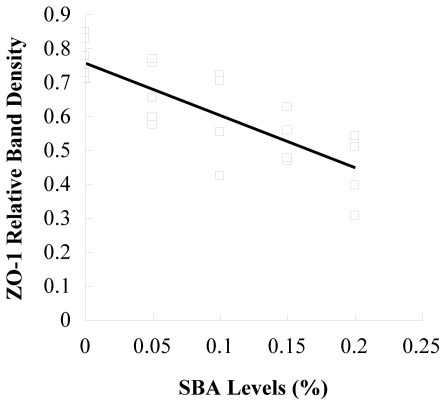
Linear correlations between ZO-1 relative band density and SBA levels. n = 5; y = −1.54x + 0.76; R^2^ = 0.592; *P* < 0.001.

The tight junction protein occludin or ZO-1 in piglets fed 0.2% SBA decreased compared with the control shown from the results of immunochemistry (Figure 3). No obvious morphological damage to the intestinal tissue was found. In Figure 4, with the increase of the SBA levels (0–0.2%), the tight junction protein occludin or ZO-1 expression declined. Occludin (*P* < 0.001) or ZO-1 (*P* < 0.001) in piglets fed 0.15 or 0.2% SBA decreased compared with the control or 0.05 SBA group. Figures 5 and 6 showed that the linear regression analysis of the occludin (*P* < 0.001, R^2^ = 0.715) or ZO-1 (*P* < 0.001, R^2^ = 0.592) expression supported a significant linear relationship among values at the different levels SBA (0–0.2%).

## 4. Discussions

Although the use of soybean meal or whole soybean products as a diet source reflect the malfunction of soybean anti-nutritional factors exhibited in previous studies to some extent, it is difficult to identify the respective role of various soybean anti-nutritional factors, such as trypsin inhibitor, SBA and allergic protein, due to the distinct anti-nutritional mechanism. Recently, with the improvement of a technique for protein purification, purified SBA has been proven to be the best option and was, therefore chosen for this study.

In the previous study, SBA caused disruption of the brush border [8]. However, the intestinal tissue of piglets fed with purified SBA was not injured in this study. This might be explained by two aspects: The purified SBA used in this study was different from the soybean protein with complex ANFs which could have lead to the intestinal damage. On the other hand, the feeding period was not long enough to induce the histopathological changes. SBA could bind to the intestinal epithelium [8]. The intestinal structure and function could be changed by the regulation of signaling molecules from the binding of SBA and epithelium. The intestinal barrier is crucial, and disruption of SBA-induced intestinal function might result in a series of negative effects.

Injured intestinal barrier increased the epithelial permeability. Diamine oxidase (DAO), as a relatively stable marker of maturation and integrity of intestinal mucosa cells, is an intracellular enzyme with high activity existing in intestinal villous cells in mammalians, especially high in the jejunum and ileum [15]. The activity of DAO in intestinal mucosa decreases when the epithelium are injured, thus DAO activity of intestinal mucosa can indicate the changes in its cellular integrity. d-lactic acid is a metabolic product of bacteria [16], present in the intestinal lumen. The intact intestinal mucosa provides a barrier function to prevent d-lactic acid infiltrating the portal blood, as an index of increase in permeability of the intestinal wall [17]. In this study, ileal mucosa DAO in piglets fed 0.1, 0.15 or 0.2% SBA decreased, and plasma d-lactic acid content was enhanced, which indicated that the intestine injured by 0.1–0.2% SBA increased the epithelial permeability. A previous study showed that SBA (90 μg/mL) slightly increased the values with quercetin glycosides or calcium ions [18], which is consistent with this study.

The apical and basolateral sides are separated by tight junctions, providing a seal between adjacent epithelial cells that restricts transport by paracellular pathway. Tight junctions are modulated in permeability by various factors, resulting in a highly dynamic transport pathway [19]. Occludin (60 kDa) was identified as the first transmembrane protein localized at tight junctions of both epithelial and endothelial cells in chicken [20] and also in mammals [21], but its precise cellular functions remain unclear. The ultrastucture of the tight junction appears unaltered and isolated intestinal tissues demonstrate the normal transepithelial resistance (TEER) in occluding-deficient mice [22,23]. However, reducing the protein level of occludin [24] enhances the paracellular permeability in a number of cell systems. ZO-1 (225 kDa), a membrane associated protein, is localized with the cytoplasmic end of occludin at tight junctions [20,25]. The increase of ZO-1 expression plays a major role in decreasing paracellular permeability [26]. It was also found in this study that the intestinal epithelial occludin or ZO-1 expression of the SBA-induced weaned piglets declined, leading to increased intestinal permeability. It suggests that the tight junction afforded by oocludin and ZO-1 is important in maintaining the epithelial barrier integrity in response to SBA.

Another finding of the current study is that the low additive levels of SBA (0–0.05%) had no significant effects on the epithelial permeability until high additive levels (0.1–0.2%) injured the gut barrier function. It suggests that SBA perturbed the integrity of the epithelium and increased the permeability of the intestinal tissue in a dose-dependent manner, which also is in agreement with results in wheat germ agglutinin [27,28].

The regression analysis reveals that SBA levels (0–0.2%) in diets show a linear relationship with the contents of DAO, D-LA, occludin and ZO-1. This indicates that increasing SBA loosened the tight junction, thus possibly providing evidence that the slack tight junction augmented the intestinal permeability. However, Pinton (2009) [29] deducted that the presence of occludin and ZO-1 alone in the tight junctions may not have been sufficient to achieve a paracellular seal in the intestinal epithelial cells. This infers that other tight junction proteins also play an important role in maintaining intestinal permeability and it is, therefore, essential to determine other relevant proteins in future studies.

## 5. Conclusions

The high dose SBA (0.1–0.2%) could increase the intestinal permeability and reduce piglet intestinal epithelial tight junction protein occludin or ZO-1 expression, while the low dose of SBA (0.05% of total diet) had no effect. The contents of DAO or d-lactic acid and the expression of occludin or ZO-1 showed a linear relationship with SBA levels (0–0.2%) in diets.

## Figures and Tables

**Table 1:** Ingredient composition and nutrient levels of the diets.

Ingredients (%)		Nutrient contents 3 (%)	
Maize	22.00	Crude protein	23.92
Fish meal	11.20	Digestive energy (MJ/kg)	16.91
Whey powder	18.00	Calcium	0.86
Milk powder	48.00	Phosphorus	0.83
Salt	0.20	Lysine	2.00
Vitamin premix 1	0.10		
Vitamin premix 2	0.20		
Limestone	0.30		

1 Vitamin premix provided per kilogram of complete diet: vitamin A, 45,000 IU; vitamin D3, 10,000 IU; vitamin E, 60 mg; vitamin K3, 5 mg; vitamin B1, 2 mg; vitamin B2, 15 mg; vitamin B6, 6 mg; vitamin B12, 0.05 mg; folic acid, 0.5 mg; biotin, 0.1 mg; niacin, 40 mg; pantothenic acid, 25 mg.

2 Mineral premix per kilogram of complete diet: Cu, 25 mg; Fe, 50 mg; Zn, 160 mg; Mn, 40 mg; Se, 0.3 mg; I, 0.3 mg.

3 Calculated value.

**Table 2:** Effects of Soybean Agglutinin on plasma d-lactic acid d-Lactate level and intestinal mucosa DAO activity in weaning piglets.

Groups	0% SBA	0.05% SBA	0.1% SBA	0.15% SBA	0.2 % SBA
DAO/(U·g^−1^)	11.69 ± 2.67 a	10.28 ± 1.54 a,b	7.97 ± 1.27 bc	7.57 ± 1.56 c	6.15 ± 1.71 c
d-lactic acid/(μg·mL^−1^)	2.11 ± 0.26 a	2.09 ± 0.29 a	2.29 ± 0.12 ab	2.45 ± 0.09 b,c	2.62 ± 0.18 c

a,b,c Means in the same row followed by different superscripts differ at the *P* values indicated. Each mean represents 5 replicates.
